# Changes in protein expression after treatment with *Ancylostoma caninum* excretory/secretory products in a mouse model of colitis

**DOI:** 10.1038/srep41883

**Published:** 2017-02-13

**Authors:** Javier Sotillo, Ivana Ferreira, Jeremy Potriquet, Thewarach Laha, Severine Navarro, Alex Loukas, Jason Mulvenna

**Affiliations:** 1 Centre for Biodiscovery and Molecular Development of Therapeutics, Australian Institute of Tropical Health and Medicine, James Cook University, Cairns, QLD, Australia; 2 QIMR Berghofer Medical Research Institute, Brisbane, QLD, Australia; 3 Department of Parasitology, Faculty of Medicine, Khon Kaen University, Khon Kaen, Thailand; 4 The University of Queensland, School of Biomedical Sciences, Brisbane 4072, Australia

## Abstract

Different reports have highlighted the potential use of helminths and their secretions in the treatment of inflammatory bowel disease (IBD) conditions; however, no reports have investigated their effects at a proteome level. Herein, we characterise the protein expression changes that occur in lamina propria (LP) and the intestinal epithelial cells (IEC) of mice with dextran sulfate sodium (DSS)-induced colitis treated with *Ancylostoma caninum* excretory/secretory (ES) products using a quantitative proteomic approach. We have shown how parasite products can significantly alter the expression of proteins involved in immune responses, cell death and with an antioxidant activity. Interestingly, significant changes in the expression levels of different mucins were observed in this study. MUC13, a mucin implicated in gastrointestinal homeostasis, was upregulated in the LP of mice with DSS-induced colitis treated with ES, while MUC2, a major component of mucus, was upregulated in the IEC. In addition, *A. caninum* proteins have an important effect on proteins with antioxidant functions and proteins involved in intestinal homeostasis and tissue integrity and regeneration. Understanding how parasites can ameliorate IBD pathogenesis can help us design novel treatments for autoimmune diseases.

Inflammatory bowel disease (IBD) comprises a group of conditions characterised by chronic inflammation of the gastrointestinal tract. Two chronic inflammatory diseases account for almost 90% of IBD cases: Crohn’s disease (CD) and ulcerative colitis (UC)^1^. Over recent years, the incidence of IBD has steadily increased with, at least, 1.4 and 2.2 million people in the United States and Europe, respectively, suffering from this disease^2^. The chronic inflammation observed in IBD is the result of an inappropriate immune response against normal intestinal flora that seems to be facilitated by defects in both the barrier function of the intestinal epithelium and the mucosal immune system^3^. The resulting inflammation of the intestinal mucosa causes abdominal pain, diarrhoea, ulcers, bloody stools and sometimes haemorrhages or cancers^4^. While CD is characterised by strong mucosal and sub-mucosal inflammation and ulceration that can occur anywhere from the mouth to the anus, the inflammation produced by UC is limited to the colon and is associated with superficial ulceration. IBD seriously impairs the quality of life of sufferers and, as it is most commonly diagnosed in people between the ages of 15 and 35, it has severe implications for the quality of life of those afflicted. It is most prevalent in developed countries and more precisely in urban areas in northern climates, with the highest incidence of ulcerative colitis in the United States and in northern European countries^2^. Despite multiple studies, the reasons and conditions for onset of these diseases are still unknown, although there seems to be a genetic cause and an undeniable effect of environmental factors and living conditions^5^,^6^.

One possible explanation for the increased prevalence of IBD in developed countries is known as the hygiene hypothesis^7^. This hypothesis suggests that the increase in autoimmune disease in developed countries is an unintended consequence of the eradication of a number of formerly widespread human pathogens, including parasitic worms such as hookworm. Over a long period of co-evolution the human immune system has adapted to the presence of these parasites and their absence can lead to the deregulation of the immune system, resulting in autoimmunity and associated conditions such as IBD^8^. The comparative absence of autoimmune disease like IBD and asthma in developing countries where parasite infections are still common^9^, adds further weight to the hygiene hypothesis.

As a corollary to the hygiene hypothesis, it has been suggested that helminth infection can protect against a range of autoimmune and/or inflammatory conditions in humans and experimental models^10^ and recent research suggests a correlation between infection with different species of helminths and a significant effect on different immune-mediated diseases, including IBD^11^,^12^. For example, *Schistosoma mansoni* infection seems to have a protective effect against anaphylaxis^13^, allergen-induced airway inflammation^14^,^15^ and also asthma in mice^16^. In preliminary clinical trials, infection with the pig whipworm *Trichuris suis* ameliorates the symptoms and pathology of Crohn’s disease and ulcerative colitis in humans^17^,^18^ and infection with *Ascaris lumbricoides* seems to reduced wheezing in humans^19^. Furthermore, infection with the human hookworm *Necator americanus* has been shown to improve gluten tolerance in patients with celiac disease and help patients with chronic CD^20^,^21^.

Hookworms produce a complex mixture of protein and lipids, known as the excretory/secretory products (ES), which are released from the surface or oral opening of the parasite. ES products are a primary interface between the parasite and the host and, as such, are most likely to contain factors important for immunomodulation in the host^22^. Many helminths induce immunoregulatory molecules that may assist them in evading host immune responses. For example, helminth infections have been shown to promote Th2 cytokines such as interleukin IL-4, IL-10 and transforming growth factor (TGF)-β, and may act to change the behaviour of pro-inflammatory immune cells such as macrophages and dendritic cells^23^,^24^. These changes could allow the parasite to evade the immune defences of its host but at the same time offer the host some protection from excessive inflammatory responses that can lead to organ damage and a decrease in the host’s life expectancy.

In this work we present a quantitative proteomics study of the protein expression changes that occur in the gut of mice with dextran sulfate sodium (DSS)-induced colitis. Furthermore, we examine the effects of ES from the model hookworm organism, *Ancylostoma caninum*, on protein expression in the same model. This analysis provides valuable information about the inflammatory pathways that are modified by parasite ES proteins and could lead to novel and more effective treatment for autoimmune diseases.

## Results

### Effect of different concentrations of *A. caninum* ES proteins on the intestine of mice with DSS-induced colitis

**Figure 1:**
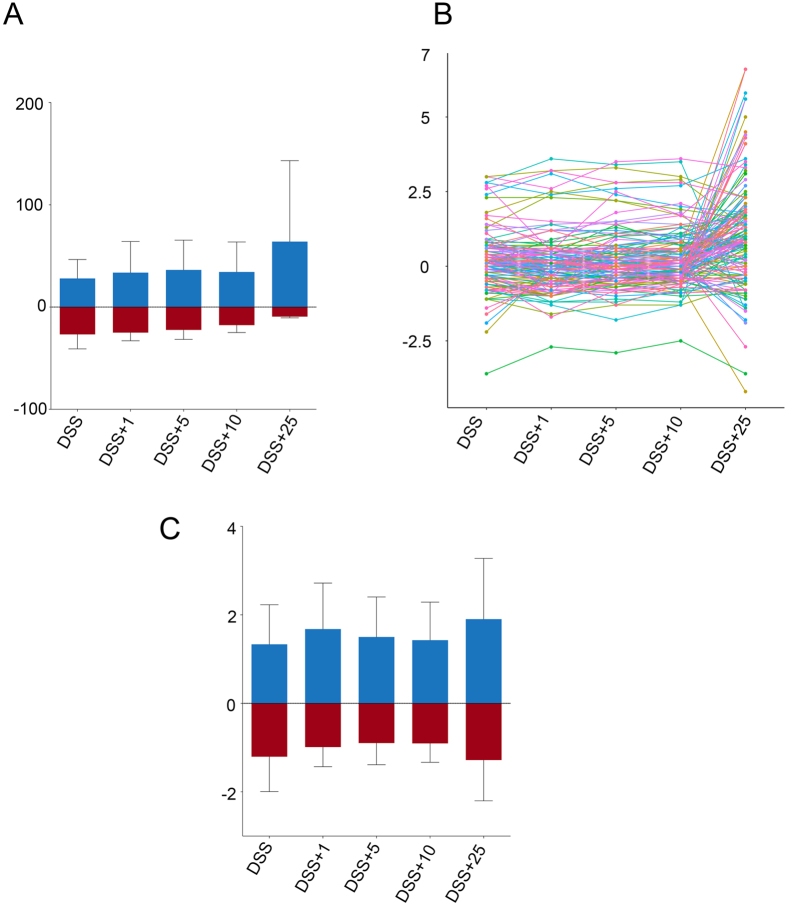
Effect of different doses of *Ancylostoma caninum* excretory/secretory (ES) proteins treatment on the expression of proteins. Number of proteins with a significantly changed expression (**A**), dot plot showing the expression changes of individual proteins (**B**) and average log_2_ fold-change of proteins with a significantly regulated expression (**C**).

Quantitative MS/MS analysis of proteins purified from the intestinal tissue of three biological replicates of treated and naïve mice yielded a total of 73,206 spectra representing 834 unique proteins (Supplementary Table 1). The effect of ES treatment on protein expression levels in the intestine of DSS-treated mice versus naïve (untreated control animals) was assessed by comparing the iTRAQ ratios of the identified proteins. After quantitative analysis, a total of 173 proteins showed significantly altered regulation after DSS or DSS+ES treatment (*P* < 0.05) (Supplementary Table 2). The number of proteins with a significantly up- or down-regulated expression, their individual expression changes and log_2_ fold-changes in DSS and DSS+ES exposed groups, relative to the naïve controls, are shown in Fig. 1A–C. DSS had a significant (*P* < 0.05) effect on the expression of 66 proteins, and the expression level of 25 of these proteins was reversed after treatment with 10 μg of ES (Supplementary Table 3, Supplementary Figure 1). Expression changes in individual proteins show a non-significant trend towards zero on the expression levels with increasing doses of *A. caninum* ES up to 10 μg, after which there was again an increase in expression ratios after treatment with 25 μg of ES (Fig. 1B,C). Mice given 10 μg of ES also had the smallest number of proteins with significantly altered expression (156 proteins among the 3 replicates) relative to the naïve group. In addition, 25 μg of ES had a great effect on proteins not previously altered by the DSS, in comparison with 1, 5 and 10 μg of ES (Supplementary Figure 2). 10 μg of ES was used in further experiments on the LP and IEC intestinal layers.

### Effect of *A. caninum* ES proteins on the LP of mice with DSS-induced colitis

To investigate the effect of *A. caninum* ES on the different constituents of the intestine, we isolated proteins from the LP of mice and performed a quantitative proteomics analysis. Samples from mice with DSS-induced colitis, treated and untreated with 10 μg of *A. caninum* ES, were compared and naïve mice treated with PBS or same amount of ES were left as controls. A total of 82,356 spectra were generated and quantified (Supplementary Table 4), which were used to interrogate the *M. musculus* proteome using Mascot and X! Tandem. After validation of protein identifications with Scaffold, a total of 1,043 proteins were identified, from which 586 contained ≥2 unique peptides. Scaffold was again used for quantification using iTRAQ reporter ions, resulting in the identification of 152 proteins differentially expressed in the DSS and DSS+ES group in relation to the controls (p < 0.05) (Supplementary Table 5). From the 152 differentially expressed proteins, 26 had been previously identified as significantly differentially expressed in the whole gut of mice with DSS-induced colitis after treatment with 10 μg of ES (Supplementary Table 6).

**Figure 2:**
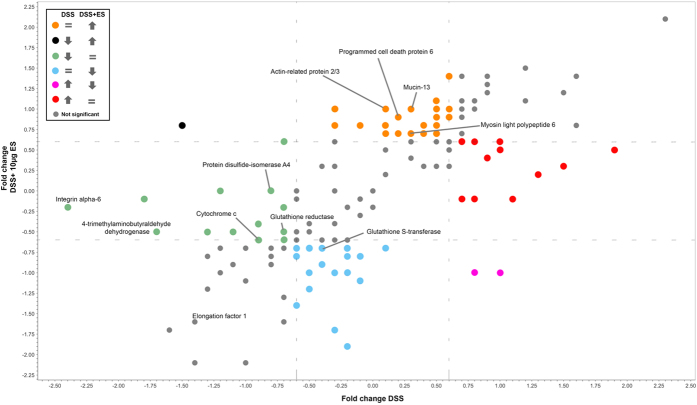
Scatter plot representing the log_2_ fold-changes of the identified proteins in the lamina propria of mice with DSS-induced colitis before and after treatment with *Ancylostoma caninum* excretory/secretory proteins. DSS and DSS+ES groups were compared against naïve group.

The effect of *A. caninum* ES on intestinal proteins was visualised by plotting the log_2_ fold-change of proteins significantly dysregulated by DSS vs. DSS+ES. Using this approach we were able to determine which proteins were dysregulated by DSS but returned to normal values after incubation with ES (red and green dots in Fig. 2), and also the proteins that were dysregulated by DSS but had a completely opposite expression after treatment with ES (pink and black dots in Fig. 2). Similarly, proteins that were significantly dysregulated only by ES (orange and blue dots in Fig. 2) were identified as proteins that could be important in the ES-mediated suppression of pathology in this mouse model of colitis.

Twenty-five proteins were up-regulated (orange dots) and 20 down-regulated (blue dots) only by *A. caninum* ES. Up-regulated proteins included proteins involved in cellular respiration (two different subunits of the cytochrome oxidase and the NADH:ubiquinone reductase), mucins (Mucin-13), proteins involved in lipid metabolism (trifunctional enzyme subunit beta) and structural proteins (myosin light polypeptide 6, actin-related protein 2/3 complex subunit 2). Down-regulated proteins included oxidoreductases (peroxiredoxin-6, glutathione s-transferase, alcohol dehydrogenase), chaperones (heat shock protein HSP 90-alpha, heat shock protein HSP 90-beta) and signalling proteins (14-3-3). Of the 69 proteins that were significantly dysregulated by DSS, 27 (11 up-regulated and 16 down-regulated) returned to normal values after the treatment with *A. caninum* ES (red and green dots in Fig. 2). Proteins initially down-regulated by DSS but restored to normal expression by ES included proteins involved in intestine integrity such as integrin alpha-6 actin, oxidoreductases and proteins involved in redox homeostasis (glutathione reductase, protein disulfide-isomerase and 4-trimethylaminobutyraldehyde dehydrogenase).

### Effect of *A. caninum* ES proteins on the IEC layer of mice with DSS-induced colitis

Proteins from the IEC layer were quantified using iTRAQ reporter ions as described above. A total of 195,996 spectra were obtained and quantified after MS/MS (Supplementary Table 7) and 1,567 proteins were identified (1,051 with ≥2 unique peptides). Of these, 292 were identified as differentially expressed (p < 0.05) in the DSS and DSS+ES group (Supplementary Table 8). From the 292 differentially expressed proteins, 44 had been previously identified as significantly differentially expressed in the whole gut of mice with DSS-induced colitis after treatment with 10 μg of ES (Supplementary Table 9).

**Figure 3:**
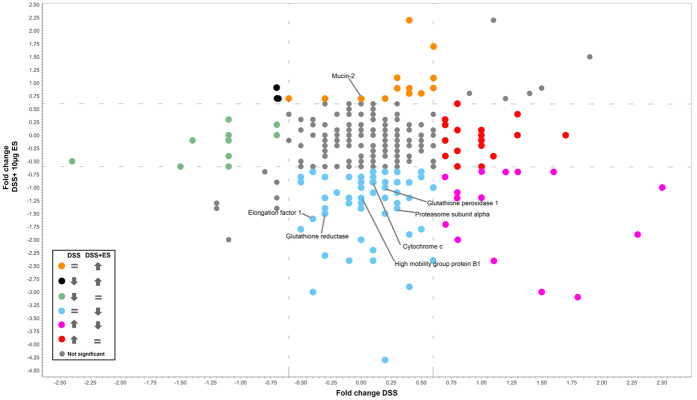
Scatter plot representing the log_2_ fold-changes of the identified proteins in the intestinal epithelial cells layer of mice with DSS-induced colitis before and after treatment with *Ancylostoma caninum* excretory/secretory proteins. DSS and DSS+ES groups were compared against naïve group.

To visualise protein expression changes caused by ES, protein expression ratios from the DSS+ES and DSS only groups were plotted against each other as described above for the LP analysis (Fig. 3). Twenty-four proteins underwent opposite regulation when the DSS and DSS+ES groups were compared to controls: 21 were down-regulated in the DSS+ES group after being up-regulated in DSS only mice (pink dots in Fig. 3), including junction plakoglobin and mitogen-activated protein kinase 3 (Mapk3); and 3 proteins, including calmodulin, were up-regulated in the DSS+ES group after being down-regulated in DSS only mice (black dots in Fig. 3). One hundred and eight proteins were only observed to be dysregulated in the DSS+ES group including proteins involved in redox metabolism (alcohol dehydrogenase NADP+, glutathione peroxidase 1, all-trans-retinol 13,14-reductase), mucins and proteins involved in mucus secretion (calcium-activated chloride channel regulator 1 and mucin-2), proteins related to energy metabolism (glyceraldehyde-3-phosphate dehydrogenase, sulphite oxidase) and proteins involved in inflammatory and immune responses (galectin-3, mitogen-activated protein kinase 1 (Mapk1) and others). Thirty proteins were significantly dysregulated in the DSS only group but returned to normal values in the DSS+ES group (red and green dots), including proteins with an oxidoreductase activity such as UDP-glucose 6-dehydrogenase and Peroxiredoxin-4.

### *A. caninum* ES regulates proteins related with homeostasis and immune response activity in the LP and IEC layer of mice with DSS-induced colitis

**Figure 4:**
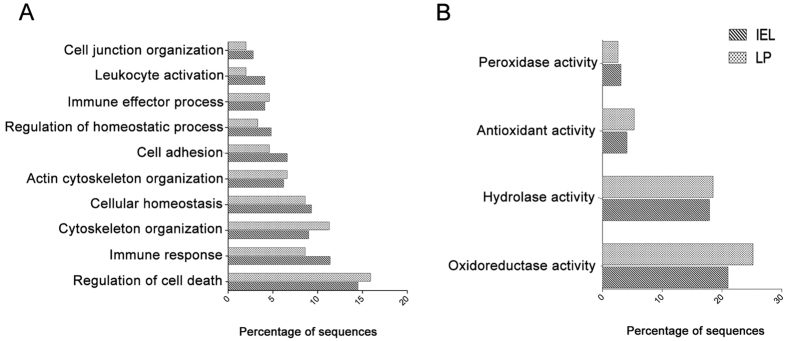
Gene ontology analysis of the proteins from the lamina propria and intestinal epithelial cells layer of mice with DSS-induced colitis with a significantly regulated expression after treatment with *Ancylostoma caninum* excretory/secretory proteins.

GO analysis was used to determine whether particular gene ontologies were over-represented by significantly dysregulated proteins. In proteins only dysregulated in the DSS+ES group the most abundant GO terms within the biological process ontology were “regulation of cell death”, “immune response”, “cytoskeleton organisation” and “cellular homeostasis” (Fig. 4A). With regards to the analysis of molecular function ontology, more than 21% and 25.2% of the proteins with a significant regulation in the IEC layer and LP (respectively) were assigned to having an oxidoreductase activity (Fig. 4B). Interestingly, when we analyse the proteins upregulated and downregulated separately, the ontologies related with the immune system (“immune system development”, “lymphocyte coestimulation” and “immune response”) were abundant in proteins with a downregulated expression in the IEL. Furthermore, we found that the antioxidant activity dominated in proteins with a downregulated expression in both the LP and IEL (Supplementary Figure 3B–D); however, the term “oxidoreductase” was mostly assigned to proteins with a significant upregulation in the LP of mice. Regarding the ontologies “cell death” and “immune system development”, they were prominent in proteins with a downregulated expression in the LP (Supplementary Figure 3C).

## Discussion

Immuno-modulatory proteins expressed by helminth parasites have great potential as therapeutics for the treatment of ulcerative colitis and Crohn’s disease, two forms of IBD that affect millions of people worldwide. Different authors have shown that the ES products from helminths can protect mice from allergic and autoimmune diseases^10^,^25^. For instance, soluble extracts from the cestode *Hymenolepis diminuta* and the nematode *Trichinella spiralis* alleviate dinitrobenzene sulfonic acid-induced colitis in a mouse model^26^,^27^; proteins from the cyst wall of the cestode *Echinococcus granulosus* have anti-inflammatory properties and protect the integrity of the intestinal mucosa in mice with DSS-induced colitis^28^; and ES products from the hookworms *Ancylostoma ceylanicum* and *A. caninum* ameliorate pathology in mice with DSS-induced colitis via the down-regulation of Th1 and Th17 responses or by inducing IL-4^+^ IL-10^+^ CD4^+^ T Cell responses respectively^29^,^30^.

IBD is characterised by a severe inflammation and disrupted homeostasis of the intestinal tissue after an abnormal activation of the mucosal immune system^3^. The LP and the IEC layer are the main mucosal immune system effectors, and failure in the balance between immunity and integrity maintenance results in mucosal homeostasis malfunction^31^,^32^,^33^. The IEC layer plays a key role in the defence against pathogens as it is in direct contact with external agents and, in the case of parasitic infection, their ES products. Moreover, the IEC layer is also in intimate contact with cells from the LP and changes in cell populations and the expression of proteins in these tissues are of importance in protection against IBD. For instance, Smith *et al*.^24^ showed that helminth infections stimulate the migration of a novel macrophage population to the LP of mice with DSS-induced colitis, suppressing colonic inflammation. Similarly, the suppression of inflammatory responses mechanistically associated with AAM*Ф*s and prostaglandins by *T. crassiceps* have a positive effect in the pathology of ulcerative colitis^34^.

Many studies have addressed the proteomic changes that occur in IBD^35^,^36^,^37^,^38^,^39^; however, no studies have analysed the effects that the parasite ES products have in its amelioration at a proteome level. Herein we have used a quantitative high-throughput mass spectrometry approach to study the changes induced by DSS in mice and the effects that the *A. caninum* ES products have on this model of colitis. We first tested the effect of different concentrations of *A. caninum* ES in the intestine of mice with DSS-induced colitis. We found that the samples from mice given a 10 μg dose of ES had the smallest number of proteins significantly differentially regulated relative to the control group. Furthermore, the effect was consistent from 1 μg to 10 μg, with the 10 μg ES sample showing the greatest effect. The highest protein ratios and number of proteins were found in the 25 μg sample, suggesting that this concentration of ES could be damaging the intestinal epithelium. Previous studies have shown that 10 and 25 μg of *A. caninum* ES induced a similar Th_2_ response in mice in the absence of DSS^29^, thus for our study we used 10 μg of ES in order to minimize tissue damage while still getting a Th_2_ response.

Ferreira *et al*.^29^ found that *A. caninum* ES could reduce the pathology in mice with DSS-induced colitis by reducing the levels of proinflammatory cytokines and recruiting M2 macrophages and eosinophils. In this work we have found different proteins involved in immune responses involved in DSS-induced colitis and ES treatment. Firstly, the expression levels of high mobility group box protein 1 (HMGB1) were significantly downregulated after treatment with *A. caninum* ES in both the LP and IEC layer tissues. Interestingly, this protein was not significantly regulated in the whole gut sample (Supplementary Table 6). This protein is secreted by different immune cells, including macrophages, and it is involved in inflammation and tissue damage via Toll-like receptor (TLR)-4 signaling^40^,^41^. Although this protein was not elevated in the DSS only group, its down-regulation in the DSS+ES group in the IEC layer might suggest an important role for this protein in inflammatory responses. Conversely, HMGB1 was over-expressed in the GS samples treated with 25 μg of *A. caninum* ES, suggesting that high levels of ES counter the anti-inflammatory effects of the lower dosages, making it unsuitable for the treatment of colitis in this model.

It is interesting to note that negative regulators of inflammatory responses, such as proteasome subunit alpha, adenosine deaminase and glutathione peroxidase 1, were also down-regulated in the IEC layer of *A. caninum* ES-treated mice. The IEC layer is composed of different types of cells such as enterocytes, goblet cells, paneth cells, tuft cells and intraepithelial lymphocytes, each having a specific function. The heterogeneous nature of this layer could make it hard to identify small effects in specific cell populations that have an important effect against IBD. Recently, an increase in the number of tuft cells has been associated with the Th_2_ responses observed after helminth infections^42^. Intraepithelial lymphocytes have been shown to play a role in homeostasis maintenance, although they can be highly influenced by microbial products, and could also contribute to IBD^32^. An integrated transcriptomic and proteomics analysis of these types of cells could shed more light on the specific function of each cell population during colitis and helminth infections.

In the present study, 54 and 29 proteins related to cell death were dysregulated in both the LP and IEC samples respectively, of which 11 were common to both tissues. In particular, most of the proteins assigned to “cell death” ontology had a downregulated ontology in both LP and IEL. The elongation factor 1, programmed cell death protein 6 (PDCD6) and cytochrome C were all down-regulated in the IEC layer of the DSS+ES group when compared to the DSS only group. Strikingly, the expression levels of PDCD6 was upregulated in the LP of the DSS+ES group while cytochrome C and elongation factor 1 were down-regulated (similar to the DSS-treated mice). IBD has been hypothesized to be an energy-deficient disease^43^, and thus down-regulation of Cytochrome C in both the IEC and LP is not surprising. PDCD6, originally found as a pro-apoptotic protein^44^, can reduce cell growth by inhibiting angiogenesis^45^. T-cell resistance against apoptosis in IBD has been suggested to be mediated by IL-12 and IL-6^46^, and Ferreira *et al*.^29^ showed how *A. caninum* ES could reduce IL-6 levels in the intestine of mice with DSS-induced colitis. Thus, reduction of IL-6 levels could enhance susceptibility to T-cell apoptosis in the LP. In this sense, the LP could be playing an important role in the restoration of the intestinal homeostasis by *A. caninum* ES products.

The analysis of significantly dysregulated proteins showed that the *A. caninum* ES has an important effect on oxidoreductases of the LP and IEC layer. A total of 60 proteins with oxidoreductase activity were dysregulated in the IEC and 38 in the LP. One of the hallmarks of IBD is oxidative stress, with reactive oxygen species (ROS) produced in high levels in the intestine. It is well known that macrophages generate and release ROS in response to different stimuli. In addition to this, M1 macrophages have been shown to activate immunopathology in IBD^47^. However, M2 macrophages play a role in protection from colitis by activating wound-healing processes in response to innate signals^48^. The extracts from different parasites (including *A. caninum* ES) has been shown to recruit M2 macrophages to the intestine of mice with induced colitis to suppress inflammation^26^,^29^. We observed a significant reduction in the expression of enzymes involved in glutathione metabolism in both the LP and IEC, in the DSS+ES group. Reductions in the level of GSH contributes to the switch from Th_1_ to Th_2_ response, which influences the shift from M1 to M2 macrophages^49^,^50^. In the IEC of the DSS+ES group the down-regulation of glutathione reductase, and other oxidoreductase proteins, could indicate a link between the shift from M1 to M2 macrophages and a low level of GSH. But in the LP this protein is already down-regulated in the DSS only group which may indicate that a more complex mechanism is operating in this layer. In this sense, other proteins with an oxidoreductase activity are upregulated in the LP, which confirms that a more complex situation is observed.

We observed a significant upregulation in the expression levels of different mucins in the LP and IEC of the DSS+ES group. For example, MUC13 was up-regulated in the LP of the DSS+ES group. The role of this mucin is uncertain as it has been linked to inflammatory and also anti-inflammatory and anti-apoptotic effects^51^,^52^, although the inflammatory properties have been related to an aberrant overexpression in gastrointestinal epithelial cells. However, MUC13 plays an important role in gastrointestinal homeostasis^52^, and it could be playing an important role in the regeneration of the intestinal barrier in the DSS+ES group. In the IEC layer, elevated levels of MUC2 were observed; however, in the whole gut the levels of MUC2 were downregulated also after treatment with ES. This mucin, produced by goblet cells, is the major component of the intestinal mucus and in addition to maintaining the epithelial barrier, it also plays a role in enhancing homeostasis by delivering tolerogenic signals critical for protection against colitis^53^,^54^. Structural proteins were also up-regulated in the LP of the DSS+ES group, including myosin light polypeptide 6 and actin-related protein 2/3 complex subunit 2. Conversely, in the DSS only group proteins involved in tissue integrity, such as integrin alpha-6, protein disulfide-isomerase and 4-trimethylaminobutyraldehyde dehydrogenase, were down-regulated. Importantly, in the DSS+ES group the expression levels of these proteins were returned to levels similar to those observed in the controls. Together, these data reinforce the hypothesis that *A. caninum* ES products are able to restore homeostasis and intestinal integrity during colitis.

In the present study we present data showing the changes that occur in the intestine of mice with DSS-induced colitis and the effect of *A. caninum* ES at a proteome level. We have shown how the parasite proteins have an important effect on proteins with antioxidant functions or playing a role in the immune system and cell death. We also observed significant changes in the expression of proteins involved in intestinal homeostasis and tissue integrity and regeneration. Hookworm secreted proteins are being screened for their anti-inflammatory properties, so understanding the mechanisms underlying the anti-inflammatory properties of ES proteins will help in the identification of novel therapeutics for the treatment of autoimmune disease.

## Methods

### Parasite material and isolation of ES products

*A. caninum* adult worms were cultured and ES products collected as described previously^29^. Briefly, parasites were incubated in serum free medium containing 100 μg of streptomycin/ml and 100 U of penicillin/μl for 24 h. The supernatant (ES) was collected, filter sterilized through a 0.22-μm-pore-size filter (Pall), and concentrated and buffer exchanged to PBS using a 10-kDa spin column (Pall). Triton X-114 (Sigma) was used to remove lipopolysaccharide (LPS) from ES as previously described^29^. Briefly, ES was incubated at 4 °C with 5% Triton X-114 for 30 min, followed by a 10 min incubation at 37 °C and a centrifugation at 1,600 *g* for 15 min at room temperature. The upper endotoxin-free phase was collected, and the process was repeated twice to ensure thorough removal of endotoxin. The protein concentration was calculated using a micro-BCA protein assay kit (Pierce), and the *Limulus* Amebocyte Lysate (Lonza) assay was used to confirm the adequate removal of endotoxin.

### Animals, DSS-induced colitis and tissue collection

The protocols used for animal experimentation were approved by the James Cook University Animal Ethics Committee. Six to eight week old female C57BL/6 mice were purchased from Animal Resource Center (Perth, Australia) and were housed and handled according to Australian animal rights and regulation standards. Mice received food and water *ad libitum*.

**Figure 5:**
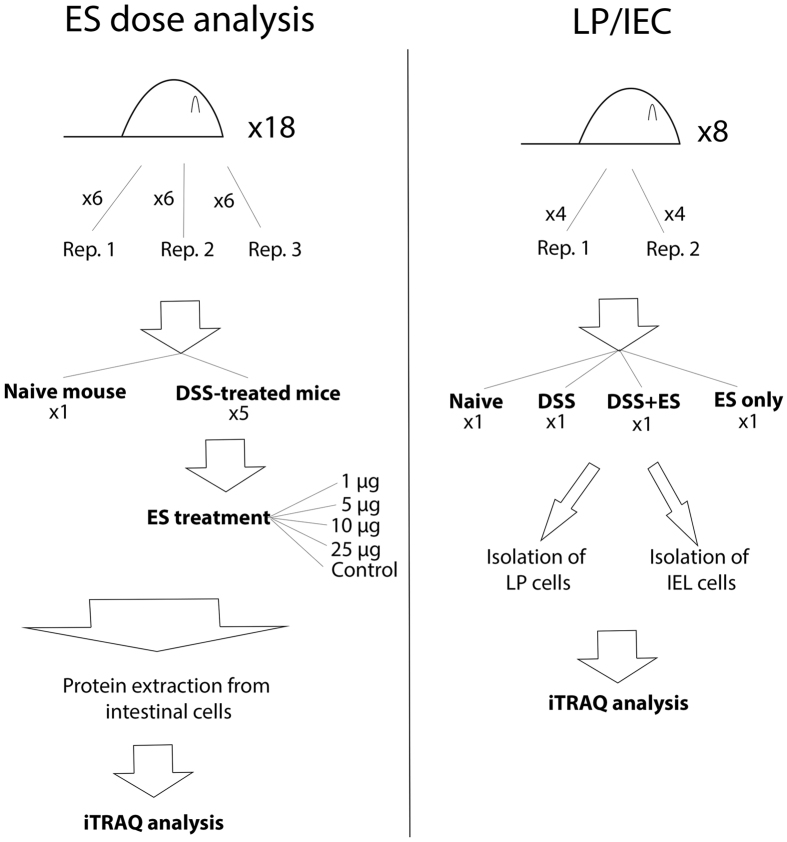
Schematic overview of the experimental strategy followed. A total of three replicates (with six mice each) were analysed to study the effect of different *Ancylostoma caninum* excretory/secretory (ES) protein doses in the intestine of mice with DSS-induced colitis (**A**). The protein changes of the lamina propria (LP) and intestinal epithelial cell layer (IEC) of mice with DSS-induced colitis treated with *A. caninum* ES proteins were analysed in duplicate (**B**).

The DSS model of colitis has been previously described^29^. Colitis was induced by administering a 3.5% (wt/vol) solution of dextran sodium sulfate (DSS 36000–50000 kDa; MP Biomedicals) to mice as a substitute for normal drinking water. To determine the dose of ES that had the greatest ameliorating effect on protein expression changes observed during DSS-induced colitis, three mice (biological replicates) per experimental condition were analysed (Fig. 5). Four animals having DSS-induced colitis received daily intraperitoneal (i.p.) injections of different amounts of *A. caninum* ES (1, 5, 10 or 25 *μ*g), and two groups of control animals (three mice with DSS-induced colitis and three healthy mice) received no ES treatment (Fig. 5). Mice were sacrificed and their intestine analysed at day 8 post DSS administration. For the proteomic analysis of the intestinal epithelial (IEC) layer and the lamina propria (LP) two replicates were performed using four groups of four mice with each group receiving either DSS only, ES only, DSS plus ES or neither DSS or ES (naïve group) (Fig. 5). The DSS only group received a mock injection of PBS instead of ES.

For the isolation of intestinal mucosa, colons were cut in 1 cm pieces and placed in a 50 ml conical tube with 5 ml of RPMI supplemented with glutamine, 5 mM EDTA, 2% fetal calf serum (FCS) and 2 mM dithiothreitol (DTT) as previously described^55^. Briefly, samples were incubated at room temperature (RT) for 30 min under agitation and filtered through a metal strainer. Tissue was collected for isolation of LP and supernatant washed with RPMI containing glutamine and 5 mM EDTA twice at 4 °C and finally pelleted for protein extraction. The tissue obtained from the previous filtration with the metal strainer was incubated in 5 ml of RPMI supplemented with glutamine, 5 mM EDTA, 2% fetal calf serum (FCS), 400 U type I collagenase and 1 mg/ml DNase I at 37 °C for 30 min under gentle agitation and filtered again through a metal strainer. Supernatant was processed as described previously and finally pelleted for LP protein extraction. IEC and LP cells were counted and the volume was adjusted for a concentration of 3 × 10^6^ cells/ml.

### Protein extraction and iTRAQ labelling

Total gut samples (GS), IEC layer and LP harvested from the colon of euthanized mice were treated with a cell lysis buffer containing 7 M urea, 2 M thiourea, 4% Chaps and 1 mM of protease inhibitor (phenylmethanesulfonyl fluoride). Lysis was performed in a high-speed shaker (Qiagen TissueLyserII), the resulting lysate was centrifuged at 8000 *g* for 5 min and the supernatants were concentrated and buffer exchanged using 10 kDa Amicon filters (Merck Millipore) retained for further analysis. iTRAQ labelling, reduction, alkylation and digestion was performed according to the manufacturer’s protocol (AB Sciex). Briefly, 100 μg of protein from each sample was denatured with 2% SDS, reduced with 50 mM Tris-(2-carboxyethyl)-phosphine (TCEP) at 60 °C for 1 h, and cysteine residues were alkylated with 10 mM methyl methanethiosulfate (MMTS) solution at RT for 10 min. Proteins were trypsin digested at 37 °C for 16 h, and each sample was labelled with different iTRAQ reagents having distinct isotopic compositions. To remove the excess of iTRAQ labeling, a HiTrap SP HP column (GE Healthcare) was used according to the manufacturer’s instructions and desalting and cleanup of samples was performed prior to electrofocusing using a Sep-Pak C18 cartridge (Waters).

### OFFGEL electrophoresis (OGE)

A 3100 OFFGEL Fractionator (Agilent Technologies) with a 24 well setup was used for pI-based peptide separation as previously described^56^. Briefly, the 24 cm long, 3–10 linear pH range IPG gel strips (GE Healthcare) were rehydrated with IPG Strip Rehydration Solution for 15 min. A total of 3.6 ml of OFFGEL peptide sample solution was used to dissolve the samples and 150 μl was loaded in each well. For isoelectric focusing, a maximum current of 50 μA was applied until 50 kVh was reached. Peptide fractions were harvested and each well rinsed with 150 μl of a solution of water/methanol/formic acid (49%/50%/1%) for 15 min. These solutions were pooled with their corresponding peptide fraction and evaporated using a vacuum concentrator. Desalting of samples was performed using ZipTip (Millipore) according to the manufacturer’s protocol followed by centrifugation under vacuum.

### Mass spectrometry and protein identification

Total gut OGE fractions were chromatographically separated on a Dionex Ultimate 3000 HPLC using an Agilent Zorbax 300SB-C18 (3,5 μm, 150 mm × 75 μm) column. A flow rate of 300 nl/min and a linear gradient of 0–80% Solvent B over 60 min was used. The mobile phase consisted of solvent A (0,1% formic acid (aq)) and Solvent B (80/20 acetonitrile/0,1% formic acid (aq)). Eluates from the RP-HPLC column were directly introduced into the NanoSpray II ionisation source of a QSTAR Elite Hybrid MS/MS System (Applied Biosystems) operated in positive ion electrospray mode. All analyses were performed using Information Dependant Acquisition, and Analyst 2.0 (Applied Biosystems) was used for data analysis. IEC layer and LP fractions separated by OFFGEL were analyzed by LC-MS/MS on a Shimadzu Prominance Nano HPLC coupled to an AB SCIEX Triple Tof 5600 mass spectrometer (Applied Biosystems). Five microliters of sample was injected onto a C18 trap column (Agilent Technologies) and desalted for 5 min using 0.1% formic acid (aq) at 30 μl/min. Peptides were then eluted onto an analytical nano HPLC column (Agilent Technologies) and separated using a 35 min gradient of 1–40% buffer B followed by a steeper gradient from 40–80% buffer B in 5 min. Buffer B contained 90/10 acetonitrile/0.1% formic acid, and buffer A consisted of 0.1% formic acid (aq). The mass spectrometer was operated in an Information Dependent Acquisition, IDA, mode, and full scan TOFMS data were acquired over the mass range 350–1400 m/z and product ion scans over the mass range of 80–1400 m/z for up to 20 of the most abundant ions with a relative intensity above 100 and a charge state of +2 –+5. Analyst TF 1.6.1 software (Applied Biosystems) was used to acquire the data.

### Bioinformatic analysis of proteomic sequence data

All MS/MS samples were analyzed using Mascot (Matrix Science, London, UK; version 2.5.1) and X! Tandem (The GPM, thegpm.org; version CYCLONE (2010.12.01.1)). Mascot was set up to search a subset of the Uniprot database containing proteins from *Mus musculus* assuming trypsin digestion. Parameters for Mascot and X! Tandem searches included a fragment ion mass tolerance of 0.60 Da and a parent ion tolerance of 0.60 Da and fixed modifications of O^+^  18 of pyrrolysine and iTRAQ8plex labelling of lysine and the n-terminus. In X! Tandem searches variable modifications specified were Glu->pyro-Glu of the n-terminus, ammonia-loss of the n-terminus, Gln->pyro-Glu of the n-terminus, oxidation of methionine, methylthio of cysteine and iTRAQ8plex labelling of tyrosine. In Mascot searches variable modifications specified were oxidation of methionine, and iTRAQ8plex labelling of tyrosine. Scaffold Q+ (version Scaffold_4.2.1) was used to validate MS/MS based peptide and protein identifications. Peptide and protein identifications were accepted if they could be established at greater than 95% and 99% probability, respectively, as specified by the Peptide Prophet algorithm^57^, and contained at least two identified peptides^58^. Proteins containing similar peptides that could not be differentiated based on MS/MS analysis were grouped to satisfy the principles of parsimony. A false discovery rate (FDR) of <0.1%, <2.1% and <2.8% for proteins from samples GS, LP and IEC layer was calculated using protein identifications validated by the Scaffold Q+ program.

Scaffold Q+ was used to quantify the isobaric tag peptide and protein identifications. Channels were corrected in all samples according to the algorithm described in i-Tracker^59^ and acquired intensities in the experiment were globally normalized across all acquisition runs. Individual quantitative samples were normalized within each acquisition run, and intensities for each peptide identification were normalized within the assigned proteins. The reference channels were normalized to produce a 1:1 fold change. All normalization calculations were performed using medians to multiplicatively normalize data. Differentially expressed proteins were determined using Kruskal-Wallis Test analysis and results expressed in log_2_ ratios. Only proteins with a *P*-value < 0.05 were taken in consideration for further analysis. Blast2GO^60^ was used to classify proteins according to gene ontology (GO) categories, and Pfam analysis was performed using HMMER v3.1b1 (http://hmmer.org/). Plots were generated using Multiplot Studio v1.5.20. The mass spectrometry proteomics data have been deposited in the ProteomeXchange Consortium (http://proteomecentral.proteomexchange.org) via the PRIDE partner repository^61^ with the dataset identifier PXD004369.

## Additional Information

**How to cite this article**: Sotillo, J. *et al*. Changes in protein expression after treatment with *Ancylostoma caninum* excretory/secretory products in a mouse model of colitis. *Sci. Rep.* **7**, 41883; doi: 10.1038/srep41883 (2017).

**Publisher's note:** Springer Nature remains neutral with regard to jurisdictional claims in published maps and institutional affiliations.

## Supplementary Material

Supplementary Figures

Supplementary Table 1

Supplementary Table 2

Supplementary Table 3

Supplementary Table 4

Supplementary Table 5

Supplementary Table 6

Supplementary Table 7

Supplementary Table 8

Supplementary Table 9
