# Supplementary material for: Changes in protein expression after treatment with Ancylostoma caninum excretory/secretory products in a mouse model of colitis
Source: Sci Rep. 2017 Feb 13;7:41883. doi: 10.1038/srep41883 (PMC5304188; doi:10.1038/srep41883)
Supplement: Supplementary Figures [file srep41883-s1.pdf]

SUPPLEMENTARY FILES

TITLE:

Changes in protein expression after treatment with *Ancylostoma caninum* excretory/secretory products in a mouse model of colitis

AUTHORS:

Javier Sotillo, Ivana Ferreira, Jeremy Potriquet, Thewarach Laha, Severine Navarro, Alex Loukas, Jason Mulvenna

FIGURE S1

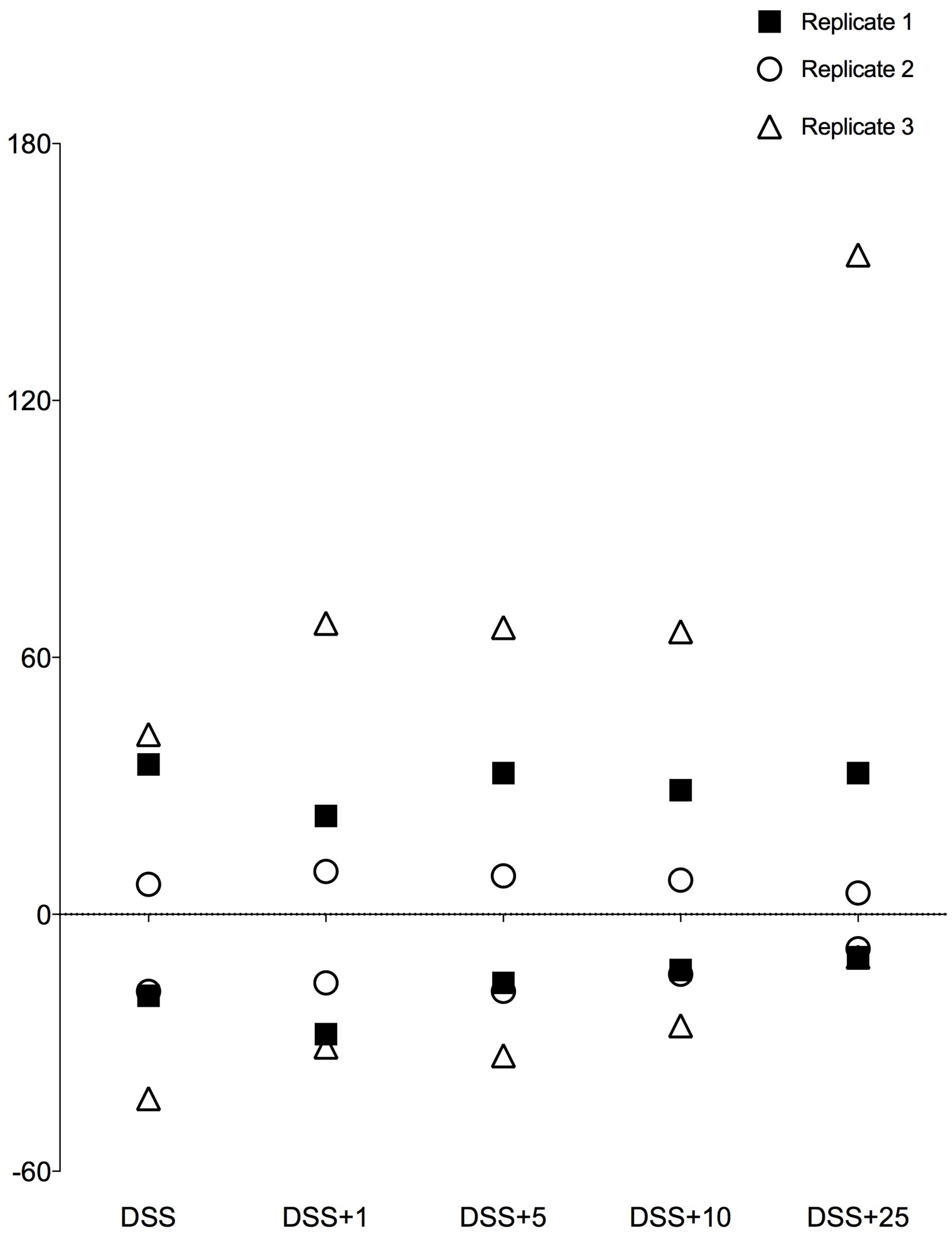

Replicate 1
Replicate 2
Replicate 3
180
120
60
0
-60
DSS
DSS+1
DSS+5
DSS+10
DSS+25

FIGURE S2

A

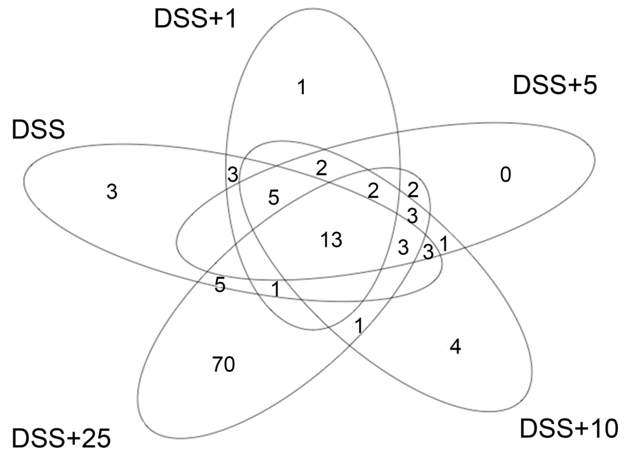

B

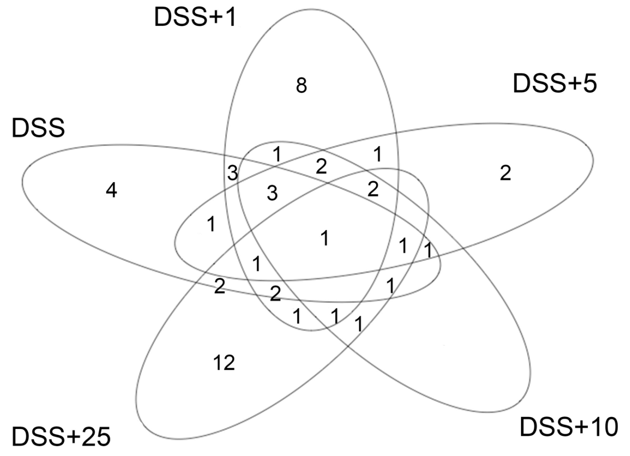

C

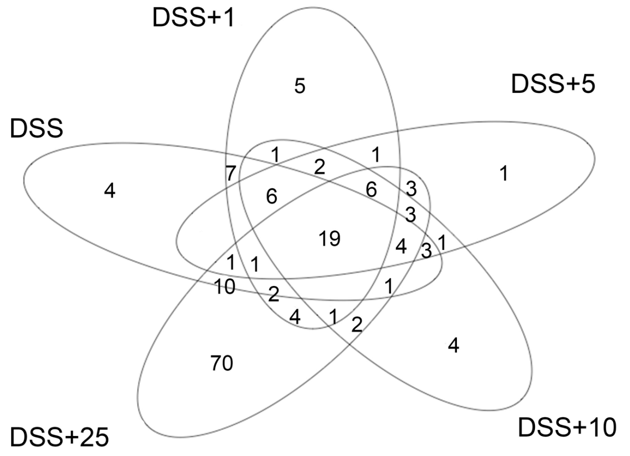

FIGURE S3

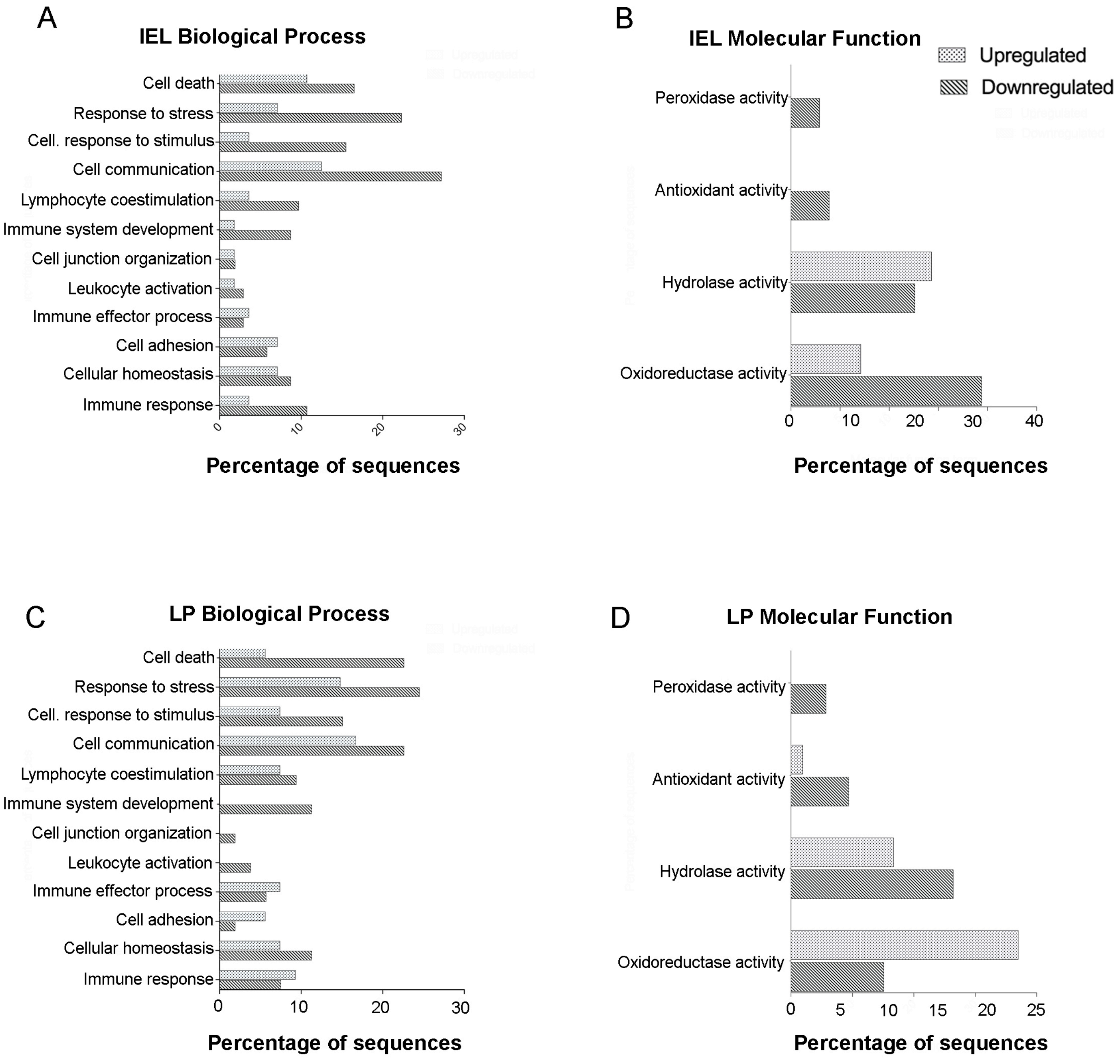
A
IEL Biological Process
Cell death
Response to stress
Cell. response to stimulus
Cell communication
Lymphocyte coestimulation
Immune system development
Cell junction organization
Leukocyte activation
Immune effector process
Cell adhesion
Cellular homeostasis
Immune response
0
10
20
30
Percentage of sequences
B
IEL Molecular Function
Upregulated
Downregulated
Peroxidase activity
Antioxidant activity
Hydrolase activity
Oxidoreductase activity
0
10
20
30
40
Percentage of sequences
C
LP Biological Process
Cell death
Response to stress
Cell. response to stimulus
Cell communication
Lymphocyte coestimulation
Immune system development
Cell junction organization
Leukocyte activation
Immune effector process
Cell adhesion
Cellular homeostasis
Immune response
0
10
20
30
Percentage of sequences
D
LP Molecular Function
Peroxidase activity
Antioxidant activity
Hydrolase activity
Oxidoreductase activity
0
5
10
15
20
25
Percentage of sequences
